# Fine needle aspiration biopsy of an osteoclast-rich undifferentiated urothelial carcinoma: A cytology case report and review of the literature

**DOI:** 10.4103/1742-6413.70407

**Published:** 2010-09-17

**Authors:** Chetna N. Purohit, Marilyn M. Bui, Ardeshir Hakam

**Affiliations:** 1 Moffitt Cancer Center, Department of Graduate Medical Education, Tampa, Florida, USA; 2 University of South Florida, Department of Pathology and Cell Biology, Tampa, Florida, USA; 3 Moffitt Cancer Center, Department of Anatomic Pathology, Tampa, Florida, USA; 4 University of South Florida, Department of Oncologic Sciences, Tampa, Florida, USA

**Keywords:** Fine needle aspiration biopsy, giant cell tumor, osteoclast-rich, undifferentiated urothelial carcinoma, urinary bladder

## Abstract

Osteoclast-rich undifferentiated carcinoma of urinary bladder (ORUCUB) is a very rare and an unusual variant of high-grade urothelial carcinoma. Here, we report an extraordinary case of metastatic ORUCUB, diagnosed by fine needle aspiration (FNA) biopsy, in a 74-year-old Hispanic male who presented with a palpable, tender left groin mass and a known previous history of high-grade carcinoma of urinary bladder and prostatic cancer. To the best of our knowledge, diagnosis of ORUCUB by FNA is the first case report in FNA cytology to be published to date. A review of the literature is emphasized on the cytological, histological and immunohistochemical features and differential diagnoses of giant cell tumor.

## BACKGROUND

Approximately 98% of the malignant tumors arising in the urinary bladder are of epithelial origin with 90% being reported as urothelial carcinoma (UC) (transitional cell carcinoma). UC occurs in predominantly older males (age 67 and older) and is three to four-times more common than in females. Most of these tumors are papillary, superficial and multifocal. They are characterized by prolonged clinical course and multiple recurrences after local resection. These papillary lesions are seen as low grade or high grade, depending on the degree of nuclear anaplasia and architectural abnormality. Low-grade non-invasive papillary UC has a low risk (<5%) of progression in comparison to high-grade non-invasive papillary UC, which has a much higher rate of progression (15–40%). The most important predictor for clinical course is depth of invasion of the tumor at the time of presentation. According to the World Health Organization, UC can have divergent differentiation, including squamous and glandular as the most common. Other less-common variants include nested, microcystic, micropapillary, lymphoepithelioma-like, lymphoma-like, plasmacytoid, sarcomatoid with or without heterologus elements, giant cell, trophoblastic, clear cell, lipid-cell and undifferentiated.

Osteoclast-rich undifferentiated carcinoma of urinary bladder (ORUCUB) is an extremely rare variant of high-grade UC and has an aggressive behavior and poor outcome. Although it has been documented in surgical pathology literature,[1–8] reports in the cytology literature are rare. However, recognizing the cytomorphological features of this rare entity is important and will be helpful in accurate diagnosis of this tumor. To our knowledge, in addition to a report of this tumor by urine cytology,[9] this is the first fine needle aspiration (FNA) cytology case report of this entity. The following case was evaluated and reported in compliance with the University of South Florida’s Institutional Review Board Policy #311.

## CASE REPORT

This case report presents a 74-year-old Hispanic male patient with a known history of high-grade carcinoma of urinary bladder and prostatic cancer, who came to the GU clinic complaining of a painful lump in the left groin for 2 weeks. Physical examination revealed a round, firm, mildly tender mass in the left upper groin. The overlying skin was intact and non-erythematous. After obtaining consent from the patient, a FNA of the mass was performed by cytopathologists using a 23-gauze needle attached to a 20-ml syringe following the standard aspiration procedure. The preliminary diagnosis was “neoplastic cells with giant cells present, final pending cellblock preparation.” The final diagnosis was “metastatic osteoclast-rich undifferentiated urothelial carcinoma.”

## MATERIALS AND METHODS

Review of the current literature revealed a total of 14 similar cases of ORUCUB. In this report, we describe the FNA cytology with the concurrent surgical case. The clinical history of the patient was reviewed. FNA cytology smears (both Diff Quick and Pap stains) and hematoxylin-eosin (HE)-stained cellblock slides were examined. Immunohistochemical (IHC) studies were performed on the cellblock with appropriate controls. The cytology and immunostains were compared with the previous transurethral resection (TUR) surgical specimen.

## RESULTS

### Cytological findings

**Figure 1:**
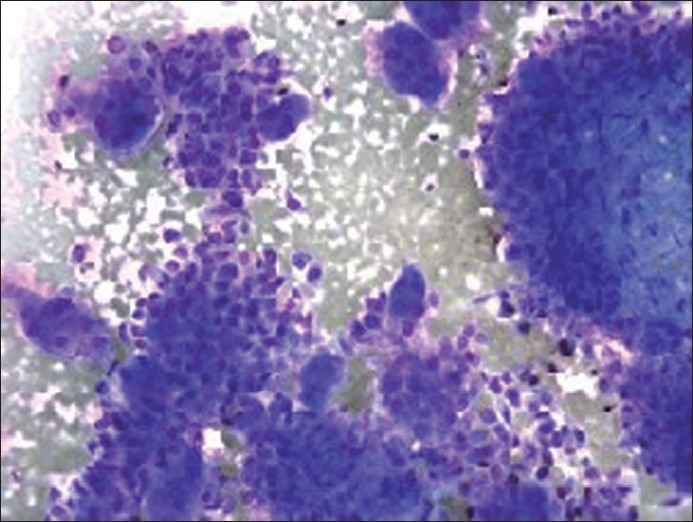
Diff Quick stain of direct smear (×200 magnification) depicting mononuclear tumor cells and osteoclast-like giant cells

**Figure 2:**
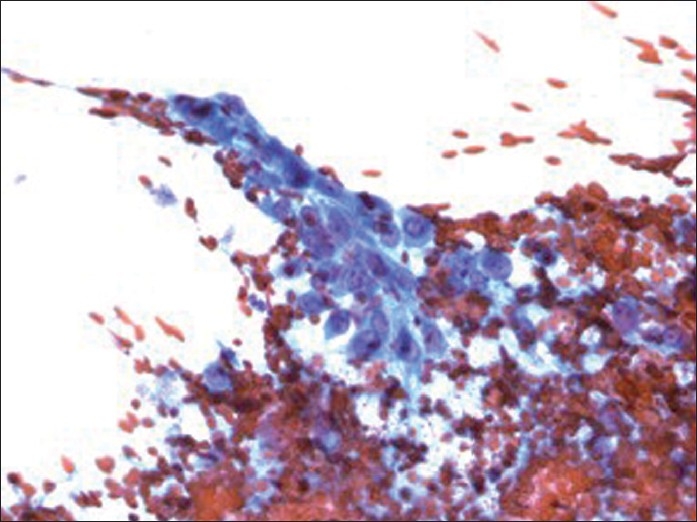
Pap stain of direct smear (×400 magnification) depicting mononuclear tumor cells with moderate cytoplasm, vesicular nuclei, high nuclear to cytoplasmic ratio and prominent nucleoli

**Figure 3:**
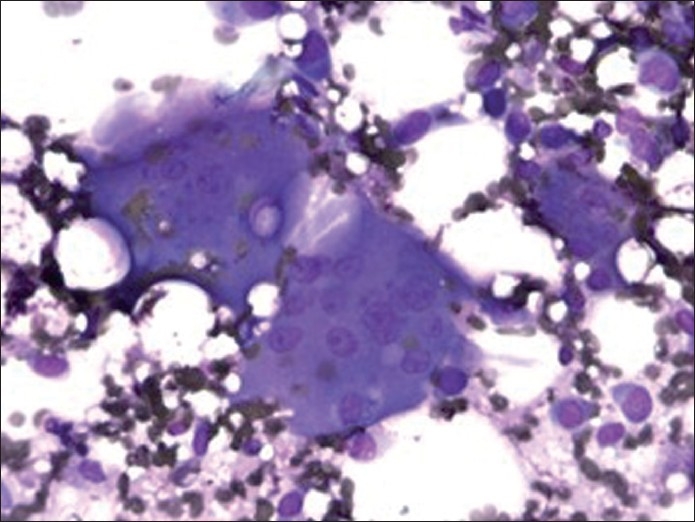
Diff Quick stain of direct smear (×400 magnification) depicting osteoclast-like giant cells

The cytological features of smears and cellblock of FNA biopsy of the left groin mass are presented in Figures 1–3. The specimen was hypercellular and consisted of highly atypical cells with two distinct populations: smaller malignant-appearing mononuclear cells in the background of abundant large benign-appearing multinucleated giant cells. The mononuclear cells were predominant. They were pleomorphic and dyscohesive. They were spindle to ovoid to polygonal shaped with moderate eosinophilic cytoplasm with large hyperchromatic to vesicular central nuclei, high nuclear to cytoplasmic ratio and conspicuous nucleoli. Few mitoses were also noted.

The giant cells were scattered and were morphologically similar to osteoclasts. They had abundant eosinophilic cytoplasm with scattered round to oval multiple nuclei (ranging from three to more than 50 in number per giant cell), showing fine and evenly dispersed chromatin, indistinct nucleoli and low nuclear to cytoplasmic ratio. No pleomorphism or mitoses were identified.

**Figure 4:**
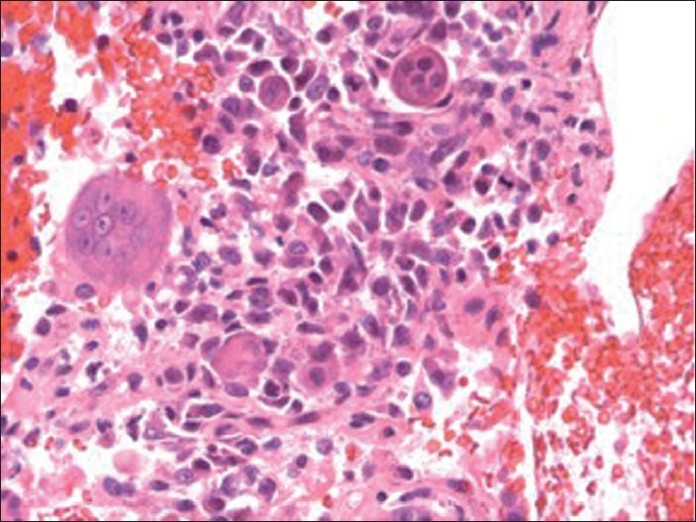
Hematoxylin and eosin stain of cellblock (×400 magnification) depicting osteoclast-like giant cells with background mononuclear tumor cells

These cytomorphologic features were indicative of high-grade UC with giant cell features. The cellblock findings [Figure 4] recapitulated the findings in the smears (air dried and alcohol fixed) and were useful in further IHC workup.

### IHC findings

**Figure 5:**
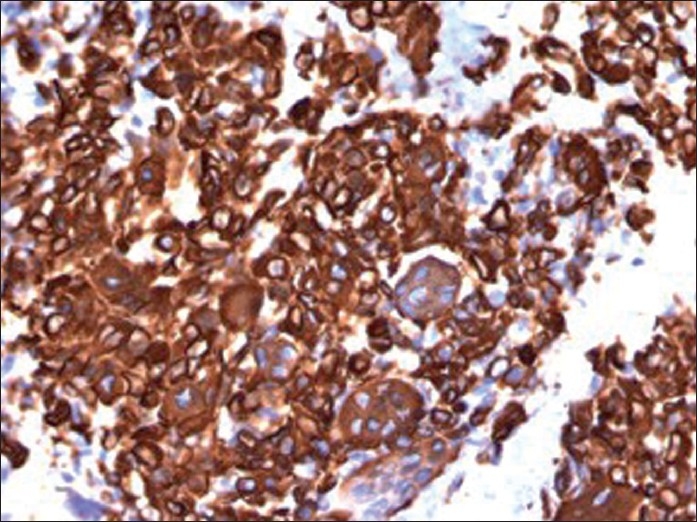
Immunostain of cellblock (×400 magnification) depicting cytoplasmic immunoreactivity with vimentin in osteoclast-like giant cells as well as in mononuclear tumor cells

**Figure 6:**
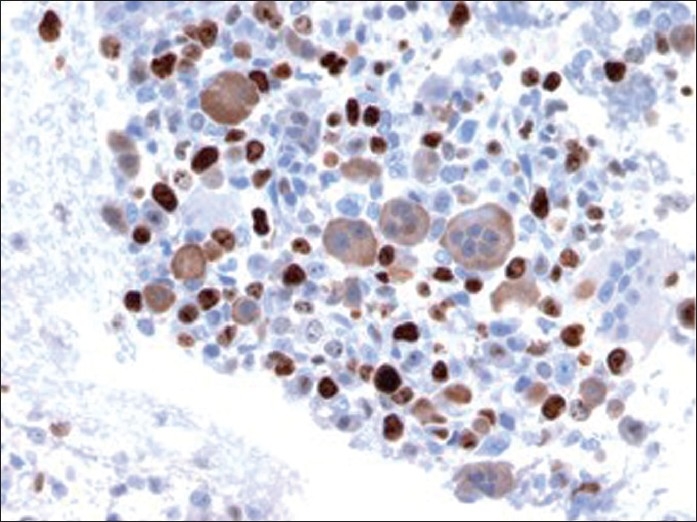
Immunostain of cellblock (×400 magnification) depicting nuclear immunoreactivity with Ki-67 in mononuclear tumor cells. Osteoclast-like giant cells are negative

**Figure 7:**
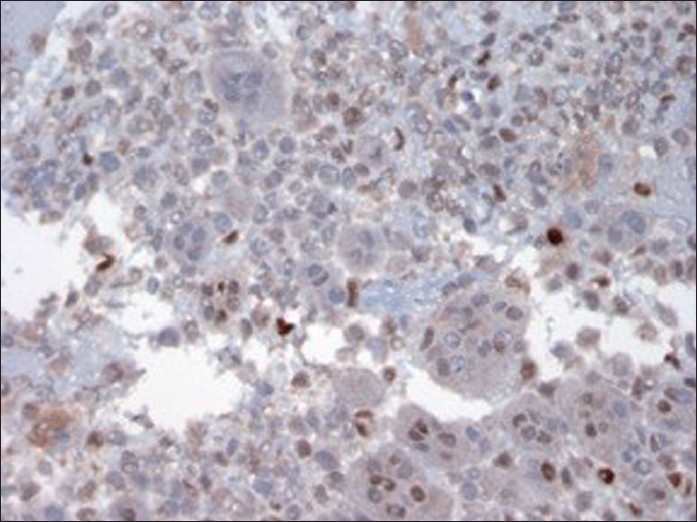
Immunostain of cellblock (×400 magnification) depicting nuclear immunoreactivity with P 53 in only background mononuclear tumor cells. Osteoclast-like giant cells are negative

**Figure 8:**
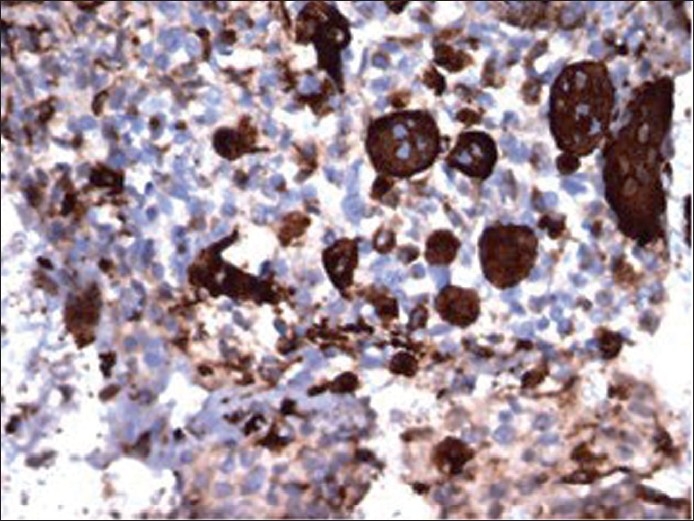
Immunostain of cellblock (×400 magnification) depicting immunoreactivity with CD-68 in osteoclast-like giant cells

IHC stains with adequate controls were performed on the cellblock [Figures 5–8]. The osteoclast-like giant cells (OCGs) showed immunoreactivity with CD-68 and vimentin but were negative for cytokeratins (CKs) (pancytokeratin cocktail, AE1, AE3, Cam 5.2, CK 5/6, CK 903, CK 7 and CK 20), epithelial membrane antigen (EMA), Ki-67 and p 53. The mononuclear tumor cells (MTCs) showed immunoreactivity with vimentin, Ki-67 and p 53 and were negative for CD-68 expression. They were also negative for pancytokeratin cocktail, AE1, AE3, Cam 5.2, CK 5/6, CK 903, CK 7, CK 20 and EMA [summarized in Table 1].

**Table 1:** Interpretation of immunohistochemical markers

*Immunostains*	*Our case surgical/cyto*	*Description*	*Implication*
AE1/AE3	Focally positive in MTCs	Keratin cocktail CK 1–8, 14–16, 10, 19	Confirm epithelial nature of tumor
Cam 5.2	Negative in MTCs/OCGs	LMK CK 8, 18–19	Confirm epithelial nature of tumor
CK 7	Negative in MTCs/OCGs	Epithelial marker 54 kDa	Confirm urothelial carcinoma (UC)
			Usually positive in a majority of UC
			Negative in urothelial adenocarcinoma (CK 7–, CK 20+)
CK 20	Negative in MTCs/OCGs	Epithelial marker 46 kDa	Apical expression in normal urothelium
			Aberrant expression (diffuse or absent staining) in UC (50%)
CK 5	CK 5/6 focally positive in MTCs	58 kDa related to CK 6	Positive (62%) in UC
CK 903 (34 beta E12)	Focally positive in MTCs	HMWK	Positive in UC (80%) and carcinoma *in situ* (diffuse expression)
EMA (epithelial membrane antigen)	Negative	CD 227/MUC1/Episialin	Positive in urothelial carcinoma
Ki-67	Focally positive in both MTCs/OCGs	Marker for cell proliferation	Determines growth fraction
P 53	Focally positive in both MTCs/OCGs	Tumor suppressor gene 53 kDa	Positive in urothelial carcinoma, associated with aneuploidy, increased S phase fraction, genetic instability
CD-68	Positive in OCGs	KPI, macrosialin	Positive in histiocytes, specific for lysosomes (macrophage/monocytes/osteoclasts etc.)
Vimentin	Positive in MTCs/OCGs	Intermediate filament for mesenchymal tissue	Widespread immunoreactivity, including endothelial cells, fibroblasts, vascular smooth muscle
SMA (smooth muscle actin)	Focally positive in MTCs	Antibody to smooth muscle actin	Positive in smooth muscle cells, myoepithelial cells etc.

Here, MTCs: mononuclear tumor cells; OCGs: osteoclast-like giant cells

### Histological findings

The histology of TUR (18 months ago) was compared with the current FNA cytology specimen from the left groin mass. Both specimens showed strikingly similar morphologic features, including the presence of multiple, evenly scattered benign-appearing osteoclast-like multinucleated giant cells in a background of ovoid–polygonal to elongated, neoplastic mononuclear cells. The surgical specimen also showed a separate fragment of superficial, high-grade papillary UC component adjacent to the OCGs and MTCs components. The lamina propria was invaded by the giant cell tumor component without invading the muscularis propria. Differential diagnosis also included sarcomatoid carcinoma.

**Figure 9:**
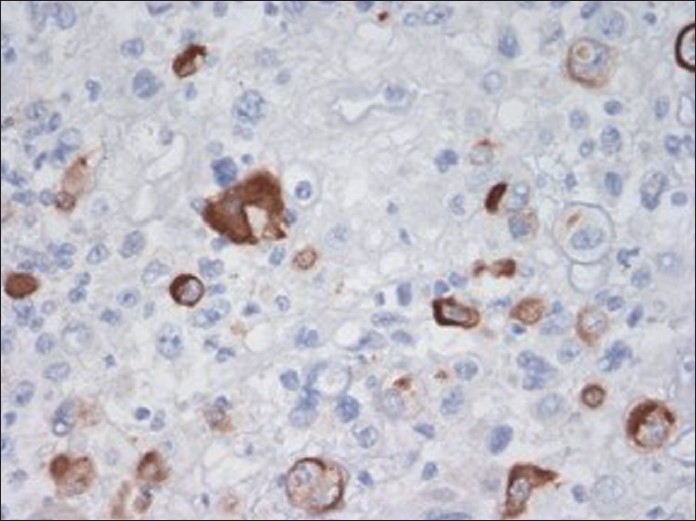
Immunostain of the surgically resected specimen (×400 magnification) depicting focal immunoreactivity with CK AE1/AE3 in mononuclear tumor cells

Following the diagnosis of FNA biopsy, the patient underwent pre-operative radiation therapy to the left groin and pelvis with concurrent chemotherapy and subsequent excision of the groin mass. Grossly, it was an irregular, ill-defined, rubbery, focally hemorrhagic and necrotic tumor measuring 5.0 cm in the greatest diameter. Histology was very similar to FNA cytology specimen, except that MTCs were more pleomorphic/anaplastic with increased number of mitoses, showing immunoreactivity for CKs (CK 903, CK 5/6 and AE1/AE3 in Figure 9), vimentin, smooth muscle actin, Ki-67 and p 53. OCGs were numerous and evenly scattered, showing immunoreactivity for CD-68 and vimentin and some focal positivity for Ki-67 and p 53.

### Clinical outcome

Despite multimodality surgical/chemo/radiotherapy, Positron emission tomography and Computed tomography PET/CT after surgery revealed progression of metastatic disease to bone, lung, lymph nodes and subcutaneous tissue, ultimately leading to the unfortunate demise of the patient, which occurred only 20 months after the diagnosis. This case again emphasizes the aggressive nature of the disease and short survival of these patients.

### Past clinical history

Retrospectively, the patient first presented with history of episodes of blood clots in the urine, without any other systemic symptoms, about 18 months ago. First TUR of his bladder lesion showed malignant neoplasm consistent with ORUCUB with adjacent fragment of high-grade papillary UC.

Pathology on subsequent two TURs and surgery (robotic-assisted laparoscopic radical cystoprostatectomy, extended bilateral pelvic lymph node dissection and ileal conduit urinary diversion was positive for conventional high-grade UC and low-grade prostatic adenocarcinoma.

## DISCUSSION

ORUCUB is an extremely rare variant of undifferentiated high-grade UC. Usually, OCG tumors arise in bone, soft tissues or tendon sheath, but tumors with a similar morphology are also reported in various visceral organs, including skin, salivary gland, larynx, thyroid, breast, lung, heart, liver, gall bladder, pancreas, intestine and female genital tract. They are known as “extra-skeletal or extra-osseous giant cell tumor,” “giant cell tumor,” “osteoclast-like giant cell tumor” or “osteoclastoma-like giant cell tumor,” etc.

Despite the morphologic similarities between giant cell tumor of bone (primary osteoclastoma) and ORUCUB, both lesions show differences in various critical aspects. Giant cell tumor of bone is a slow-growing, benign but locally aggressive neoplasm of the epiphysis/metaphysis of long bone occurring in young adults with a slight female predominance. It typically shows neoplastic monomorphic, ovoid to polygonal or elongated mononuclear cells with uniformly interspersed large OCGs. Malignant transformation is very rare (<1% and can occur after radiation therapy). However, ORUCUB is malignant neoplasm of predominantly older males, with very aggressive behavior and poor prognosis, including extensive metastasis and short survival. This tumor is considered a variant of high-grade UC because, usually, it is intimately associated with conventional high-grade UC (carcinoma *in situ*, high-grade papillary UC or both in adjacent areas of the same specimen). It is composed of two types of cells, including pleomorphic (ovoid–polygonal or spindle shaped), mononuclear malignant epithelial cells expressing immnoreactivity for epithelial markers, e.g. CKs, which is identical to adjacent conventional UC, and benign-appearing, evenly dispersed OCGs, which morphologically appear similar to osteoclasts/macrophages with multinucleation. The latter has no mitoses, some have phagocytosis and are ultrastructurally similar to osteoclasts. They do not display any epithelial marker immunoreactivity (including CKs and EMA) and are diffusely positive for osteoclast/macrophage lineage markers, including CD-68, CD 51, CD 54 and, sometimes, leukocyte common antigen. They are benign and are thought to be reactive in origin.

To date, there are 14 cases of ORUCUB[1–9] reported in the current literature. To the best of our knowledge, this is the first FNA cytology case report of ORUCUB to be published.

Making the diagnosis of an ORUCUB is important on a cytology specimen. The unusual stromal reaction of non-neoplastic, multinucleated, OCGs in a background of neoplastic mononuclear cells can be confused with so many benign and malignant entities. The differential diagnoses include giant cell carcinoma of the bladder, foreign body/granulomatous reaction, trophoblastic variant of UC, sarcomatoid carcinoma of bladder, pleomorphic giant cell carcinoma of prostate, sarcomatoid carcinoma of prostate, chemo/radiation therapy-induced giant cells in a treated prostate cancer and giant cell tumor of bone.

ORUCUB is different from giant cell variant of UC in which giant cells are epithelial and are neoplastic, showing nuclear pleomorphism and immunopositivity with CKs. ORUCUB, as mentioned earlier, has giant cells that are of osteoclast/macrophage lineage and are benign/reactive in nature, showing nuclear monomorphism and expressing immunoreactivity with CD-68 and other osteoclastic markers. In the trophoblastic variant of UC, which shows trophoblastic giant cells (syncytiotrophoblast), staining is positive for human chorionic gonadotropin. Trophoblastic differentiation is usually considered a more aggressive variant of UC rather than a germ cell tumor[10]

Sarcomatoid carcinoma of bladder with or without heterologus elements is another variant of high-grade UC, which is a biphasic malignant neoplasm showing morphological and immunohistochemical evidence of both epithelial and mesenchymal differentiation. The epithelial component is usually urothelial, glandular or small cell component showing immunoreactivity for CKs, but the mesenchymal component is frequently an undifferentiated high-grade spindle cell neoplasm showing reactivity with vimentin. Both components can be segregated from each other, but histological transition between the two components is usually noted. Both spindle and giant cell components are overtly malignant and stain with epithelial markers. In the foreign body type or granulomatous reaction of urinary bladder, the giant cells are foreign body type or Langerhan’s type with non-neoplastic plump stromal cells. They lack the malignant-appearing mononuclear cells.

Our patient was also found to have conventional prostatic adenocarcinoma. Therefore, pleomorphic giant cell carcinoma of the prostate could be a differential diagnosis, in which the multinucleated giant cell component is very bizarre and anaplastic (same as giant cell carcinoma of other organs). Prostatic adenocarcinoma and pleomorphic giant cells, both variably express prostate-specific antigen and CKs.[11,12] However, the possibility of metastatic pleomorphic giant cell carcinoma of prostate or metastatic sarcomatoid carcinoma of prostate was ruled out due to the benign appearance of the giant cells. Radiation therapy is reported as etiologic factor for giant cell carcinoma of urothelium, sacromatoid carcinoma of prostate[13] and as local therapy effect in an orthotopic model in the form of tumor necrosis, fibrosis, multinucleated giant cell induction with condensed nuclei, apoptosis and dense eosinophilic cytoplasm.[14] Our patient did not have any history of getting any radiation therapy prior to his ORUCUB.

It is known for giant cell tumor of bone to metastasize. However, in this case, there is no clinical history of bone tumor. In addition, the presence of malignant mononuclear cell is not inconsistent with a giant cell tumor of bone.

Based on the current literature, the clinical, histological and immunohistochemical features of this tumor were published in 14 cases, including one urine cytology case, and are summarized in Table 2. In almost all cases, the surface urothelium showed a non-muscle invasive UC component (either carcinoma *in situ* or high-grade papillary UC or both with or without lamina propria invasion). The UC component was focal and situated adjacent to or was overlying the giant cell tumor component (neoplastic mononuclear ovoid stromal cells and scattered reactive OCGs).

**Table 2:** Literature review of osteoclast-rich giant cell urothelial carcinoma of urinary bladder

*Ref. no./TC*	*Case no.*	*Age and sex*	*Treatment*	*Associated UC or CIS or prior history*	*Histology similar to giant cell bone tumor*	*Immunohistochemistry*		*Clinical outcome*
						*MTC*	*OCG*	
1/3	1	81 M	TUR	Y	Necrosis, mild pleomorphism	CD-68	CD-68, LCA, CD 51 and 54	NA
	2	81 M	TUR	Y	No necrosis, mild pleomorphic and atypical mitoses	CD-68, actin, desmin	CD-68, LCA, CD 51 and 54	R UC (4 months)
	3	67 M	RC	Y	Necrosis, moderate pleomorphism and atypical mitoses	EMA, LCA S-100, actin, CD-68	CD-68, LCA, CD 51 and 54	Deceased (12 months)
2/2	4	74 M	TUR	Y	Plump spindle cells in whorled pattern, mitoses	Vim, TRAP	Vim, TRAP muramidase	NA
	5	69 M	TUR	Y	Same morphology as case 4	Vimentin, TRAP	Vimentin, TRAP	NA
3/1	6	67 M	TUR	Y	Vascular stroma, permeation of vessels by giant cells	AP, PAS AAT	AP, PAS	RMF (17 months)
4/1	7	73 F	TUR, APE, V	N	Same morphology as giant cell bone tumor	NA		R 1 month TUR, RMF (6 months)
5/2	8	65 M	TUR	Y	Same morphology as giant cell bone tumor	NA	Vim, AP	RMF
	9	75 F	RC	Y	Same morphology as giant cell bone tumor	NA	Vim, AP	RMF
6/1	10	60 F	TUR	Y	Abundant histiocytes	NA	TRAP, CD-68	RMF (12 months)
7/1	11	62 M	TUR, RCP w/PU	Y	Marked tumor necrosis	NA	CD-68, Vim VIII, CD 31, S 100	RMF (5 months)
8/2	12	56 M	TUR	Y	Vascularized stroma, extravasated RBCs, blood lakes, minimal atypia	CD-68, Vim SMA Ki-67, P 53	CD-68, Vim LCA, TRAP	RMF (43 months)
	13	74 M	TUR	Y	Same as 12	CD-68, Vim Ki-67, P 53	CD-68, Vim LCA, TRAP	RMF (50 months)
9/1	14	63 M	TUR	N	Background of acute inflammation and abundant RBCs	CK 903, thrombomod-ulin	Not described	No follow-up (16 months)
Our case/1	15	74 M	TUR, TURR, RCP	Y	Necrosis, marked pleomorphism and mitoses	Vim, CK, Ki-67, P 53	CD-68, Vim Ki-67, P 53	Deceased (20 months)

Here, M: male; F: female; TUR: transurethral resection; TURR: transurethral re-resection; N/A: not available; Y: Yes; N: No; RC: radical cystectomy; RCP: radical cystoprostatectomy; PU: partial ureterectomy, APE: anterior pelvic exenteration; V: vaginectomy; RMF: recurrence and metastasis free; R and M: recurrence and metastasis; Vim: vimentin; AP: acid phosphatase; TRAP: tartarate-resistant AP; LCA: leukocyte common antigen; AAT: alpha 1 anti-trypsin.

ORUCUB is considered an osteoclast-rich undifferentiated UC (a variant of high-grade undifferentiated UC) due to the intimate association of this tumor with high-grade conventional UC and similar IHC profile, including positivity with CKs, EMA, Ki- 67 and p 53 in neoplastic mononuclear cells as well as in the adjacent UC component with non-neoplastic reactive histiocytic lineage of OCGs that are CD-68 positive and Ki-67/p 53 negative. In our case, left groin mass excision showed neoplastic mononuclear cells to be markedly pleomorphic with bizarre nuclei and increased mitoses, expressing focal positivity for CKs AE1/AE3, CK 903 and CK 5/6 and multinuclear giant cells to be focally positive for Ki-67 and p 53. As described previously, these OCGs are seen in the background stroma due to benign immunological reaction, and their presence is not specific for ORUCUB. They can be seen in a variety of benign and malignant conditions of various organs. Some studies clearly identify a number of common factors necessary for the multinucleation (cell fusion), including vitronectin, an adhesion protein, dendritic cell-specific transmembrane protein (DC-STAMP), a fusion factor, and macrophage fusion receptor that contribute to giant cell formation and function. These multinucleated giant cell phenotypes vary depending on their local environment.[15] The osteoclast is hematopoetic in origin, derived from monocyte–macrophage lineage cells that fuse to form a multinucleated osteoclast. Both locally produced cytokines and systemic hormones regulate normal osteoclast formation. The bone microenvironment plays a critical role in osteoclast formation. Marrow stromal cells or osteoblasts produce osteoclast differentiation, inducing factors that are receptor activators of the NF-kappaB ligand (RANKL), a member of the tumor necrosis factor (TNF) gene family. This factor is also called TRANCE or osteoprotegerin ligand. RANKL activity can be blocked by the soluble decoy receptor, osteoprotegerin (OPG/osteoclastogenesis inhibitory factor, a member of TNF family). The ratio of RANKL to OPG regulates osteoclast formation and activity. In neoplasia, several factors including IL-1, IL-6, PTHrP, RANKL and MIP-1alpha, have been implicated in osteoclast formation and bone destruction. PTHrP is the major factor produced by breast cancer cells that induce osteoclast formation through upregulation of RANKL. Enhanced RANKL expression is also implicated in bone destruction in multiple myeloma patients.[16] OCGs are frequently seen in tenosynovial giant cell tumor, pigmented villonodular synovitis and giant cell tumor of bone. They are CD68 -postive monocytic cell lineage composed of predominantly macrophages recruited in response to macrophage colony-stimulating factor 1 (CSF1).[17] Understanding of various molecular mechanisms responsible for osteoclast activation and osteoclast-like cell formation in certain neoplasms should lead to novel therapeutic approaches.[17,18] However, due to the rarity of ORUCUB, the mechanism of multinucleated giant cell formation has not been investigated. In our case, it is difficult to say whether these giant cells are still benign/reactive or are of uncertain malignant potential. The fact that the patient expired 2 months later makes us speculate that the positivity in p 53 and Ki-67 may be associated with advanced disease course, aggressive behavior and bad clinical outcome. In our case, the CK stains were negative in MTCs on cellblock on comparing with the surgical specimen, which was positive for CK. This may be the result of tumor heterogeneity and limited nature of sampling by FNA. Prior clinical history and comparison with prior resection specimen was helpful in making a definitive diagnosis of ORUCUB.

Molecular testing in the diagnosis of conventional UC is promising. The role of fluorescence *in situ* hybridization (FISH) such as Urovision is well established.[19,20] Urovision FISH is designed to perform testing on cytospin slides prepared from fresh urine specimen. In the past, no Urovision study was carried out on our patient.

There are few studies reported on the activities and effects of MAPKs pathway,[21,22] including MEK, ERK½, ELK and P38 in normal urothelial cells and UC. However, these studies used samples of the most common and conventional types of UC. Because ORUCUB is a rare variant of UC, no molecular study of this particular variant is reported so far.

## CONCLUSION

Metastatic ORUCUB is a rare but specific diagnosis that can be recognized by FNA cytology.

## COMPETING INTEREST STATEMENT BY ALL AUTHORS

No competing interest to declare by any of the authors.

## AUTHORSHIP STATEMENT BY ALL AUTHORS

Each author acknowledges that this final version was read and approved. All authors qualify for authorship as defined by ICMJE http://www.icmje.org/#author Each author participated sufficiently in the work and takes public responsibility for appropriate portions of the content of this article.

## ETHICS STATEMENT BY ALL AUTHORS

Our institution does not require approval from the Institutional Review Board (IRB) for a case report without identifiers.

## EDITORIAL / PEER-REVIEW STATEMENT

To ensure integrity and highest quality of CytoJournal publications, the review process of this manuscript was conducted under a double blind model(authors are blinded for reviewers and reviewers are blinded for authors)through automatic online system.
